# The mediating role of smartphone addiction in the relationship between desire for being liked and psychological resilience

**DOI:** 10.3389/fpsyg.2026.1922313

**Published:** 2026-10-29

**Authors:** Ahmet Yavuz Mallı, Ozkan Isik, Yunus Şahinler, Laurentiu-Gabriel Talaghir, Florentina Cristea, Ștefan Cristian Liușnea

**Affiliations:** 1 Faculty of Sport Sciences, Erzincan Binali Yıldırım University, Erzincan, Türkiye; 2 Faculty of Sport Sciences, Balıkesir University, Balıkesir, Türkiye; 3 Faculty of Sport Sciences, Istanbul Gelisim University, Istanbul, Türkiye; 4 Faculty of Physical Education and Sport, Dunarea de Jos University of Galati, Galati, Romania

**Keywords:** desire for being liked, mediation model, psychological resilience, smartphone addiction, sports sciences students

## Abstract

**Purpose:**

This study examined the association between the desire for being liked and psychological resilience among university students enrolled in faculties of sports sciences in Türkiye and investigated whether smartphone addiction statistically mediated this association.

**Method:**

A cross-sectional correlational design was employed. Participants were 237 university students (159 males and 78 females) enrolled in faculties of sports sciences in Türkiye, recruited using a convenience sampling method. Data were collected using the Desire for Being Liked Scale, the Smartphone Addiction Scale–Short Version, and the Brief Resilience Scale. Pearson correlation analysis and a bootstrap mediation analysis (PROCESS Macro Model 4; 5,000 resamples) were conducted to examine the indirect association between the variables. Gender was included as a covariate in the mediation analysis due to the unequal distribution of male and female participants in the sample; its inclusion did not materially alter the estimated direct or indirect effects.

**Results:**

The desire for being liked was positively associated with smartphone addiction (*B* = 0.713, *p* < 0.001), whereas both the desire for being liked (*B* = −0.137, *p* = 0.004) and smartphone addiction (*B* = −0.066, *p* = 0.006) were negatively associated with psychological resilience. The indirect association between the desire for being liked and psychological resilience through smartphone addiction was statistically significant (indirect effect *B* = −0.047, 95% bootstrap CI [−0.094, −0.011]). The observed covariance pattern was compatible with the proposed mediation model; however, the final model explained a modest proportion of the variance in psychological resilience (*R*^2^ = 0.099).

**Conclusion:**

The findings indicate that a stronger tendency to seek social approval was associated with higher levels of smartphone addiction and lower levels of psychological resilience. Smartphone addiction showed a statistically significant indirect association in the relationship between the desire for being liked and psychological resilience. Given the cross-sectional design and the modest explanatory power of the model, these findings should be interpreted as associations rather than causal effects and may contribute to understanding psychosocial adjustment among university students in sports sciences.

## 1 Introduction

As digital technologies have become an integral part of everyday life, substantial increases have been observed in individuals’ levels of smartphone use and in related behavioral addiction tendencies (Kuss and Griffiths, 2017; Özbek et al., 2026). The widespread adoption of smartphones and digital communication technologies has also changed the ways in which individuals maintain social relationships and seek interpersonal interaction. Although digital platforms may facilitate opportunities for social connection and communication, excessive smartphone use has been associated with a range of psychosocial problems, including smartphone addiction, depression, anxiety, loneliness, and reduced psychological wellbeing (Billieux et al., 2015; Birkok et al., 2026; Elhai et al., 2017; Genç and Genç, 2026; Koç et al., 2026; Samaha and Hawi, 2016). Among university students, who constitute one of the most active groups of smartphone users, problematic smartphone use has attracted increasing attention because of its potential implications for psychological functioning and adjustment (Gökçearslan et al., 2016). Within this context, examining psychological characteristics that may be associated with problematic smartphone use and psychological resilience has become an important area of research. Students enrolled in faculties of sports sciences represent a population for whom psychological resilience is particularly important because of the academic, physical, and competitive demands associated with their education and future professional roles (Fletcher and Sarkar, 2012). Compared with many other university students, they are frequently exposed to performance evaluations, competitive environments, and social interactions in which physical competence and athletic performance are highly valued (Brewer et al., 1993; Sabiston et al., 2019). These contextual characteristics may increase the relevance of psychosocial factors related to interpersonal acceptance, self-evaluation, and adaptive coping. However, it should be noted that the present study did not directly assess variables such as body image, athletic identity, or self-presentation. Therefore, these characteristics are presented only as contextual features frequently discussed in the literature rather than as measured determinants of the observed associations. Within this context, examining whether the desire for being liked, smartphone addiction, and psychological resilience are statistically associated may contribute to a better understanding of psychosocial functioning among university students enrolled in faculties of sports sciences.

Self-Determination Theory (SDT) provides one of the strongest theoretical frameworks for explaining individuals’ orientation toward digital environments (Deci and Ryan, 2000). According to SDT, human behavior is guided by the fulfillment of three basic psychological needs: autonomy, competence, and relatedness. Among these needs, relatedness reflects individuals’ desire to establish meaningful interpersonal relationships and to experience acceptance and belonging within their social environment (Przybylski et al., 2013). From this perspective, the desire for being liked can be conceptualized as a general interpersonal tendency reflecting individuals’ motivation to obtain social acceptance and positive evaluations from others rather than as a specifically digital or social media–based construct. Individuals with stronger approval-seeking tendencies may therefore be more inclined to engage in frequent smartphone-mediated social interactions, as smartphones provide convenient and continuous opportunities for interpersonal communication. Although the present study did not directly assess digital approval-seeking or social media feedback behaviors, previous studies have suggested that individuals with stronger interpersonal approval needs may be more vulnerable to problematic smartphone use (Casale and Fioravanti, 2017; Lee and Sung, 2016). Accordingly, SDT provides a theoretical rationale for examining whether the desire for being liked is statistically associated with smartphone addiction.

The Uses and Gratifications Theory, which explains individuals’ motivations for digital media use, argues that individuals actively select media tools to satisfy specific needs (Katz et al., 1973). From this perspective, smartphones represent multifunctional tools that facilitate communication, access to information, entertainment, and interpersonal interaction. Individuals whose psychological needs for social connection and interpersonal acceptance are relatively stronger may therefore rely more heavily on smartphone-mediated communication as one of several available means of maintaining social relationships (Whiting and Williams, 2013). Frequent reliance on smartphones for these purposes may be associated with maladaptive patterns of smartphone use among some individuals (Kuss and Griffiths, 2017). Importantly, the present study did not directly examine specific smartphone activities, such as social media use, online approval seeking, or feedback-monitoring behaviors. Therefore, the proposed association between the desire for being liked and smartphone addiction is interpreted at the level of general smartphone use rather than any specific digital behavior.

Social Comparison Theory offers an important perspective for explaining the psychological resilience dimension of the present study (Festinger, 1954). Individuals tend to compare themselves with others in order to evaluate themselves, and social media provides an unlimited field of data for this process (Vogel et al., 2014). Social media environments intensify social comparison processes by constantly exposing individuals to the selectively presented lives and achievements of others (Fardouly et al., 2015). Excessive engagement with smartphone-based content may expose individuals to repeated comparison experiences, information overload, and cognitive-emotional demands, all of which have been associated with reduced psychological wellbeing and adaptive coping in previous research. Consequently, problematic smartphone use may be statistically associated with lower psychological resilience, particularly in the context of difficulties in coping effectively with everyday stressors (Smith et al., 2008). Because the present study did not measure social comparison directly, this theoretical explanation should be interpreted as one possible interpretation of the observed associations rather than as an empirically tested pathway.

Taken together, these theoretical perspectives provide a coherent rationale for the proposed mediation model. SDT suggests that individuals with stronger interpersonal approval-seeking tendencies may place greater importance on social acceptance. The Uses and Gratifications Theory further proposes that such individuals may rely more heavily on smartphone-mediated communication to satisfy interpersonal and psychological needs, which may be associated with greater vulnerability to problematic smartphone use. Finally, Social Comparison Theory offers a possible theoretical perspective on the observed association between problematic smartphone use and lower psychological resilience, particularly in relation to individuals’ exposure to psychologically demanding comparison processes and their adaptive coping resources. Accordingly, smartphone addiction was positioned as a potential statistical mediator in the association between the desire for being liked and psychological resilience.

At the same time, alternative theoretical explanations are also plausible. For example, individuals with lower psychological resilience may be more likely to rely on smartphones as a coping strategy or may become increasingly dependent on external social approval. Because the present study employed a cross-sectional design, the temporal ordering and causal direction of the associations among these variables cannot be established. Accordingly, the direct, indirect, and total effects estimated in the mediation model should be understood as statistical associations rather than causal effects. Therefore, the proposed mediation model should be interpreted as one theoretically informed explanation that is compatible with the observed pattern of associations rather than as evidence of causal relationships.

Although previous studies have examined the bivariate associations among these variables, few studies have investigated their statistical interrelationships within an integrated mediation model among university students enrolled in faculties of sport sciences. Accordingly, the present study aimed to examine the associations among the desire for being liked, smartphone addiction, and psychological resilience and to investigate whether smartphone addiction statistically mediated the association between the desire for being liked and psychological resilience among university students enrolled in faculties of sport sciences in Türkiye. In line with this aim, the hypotheses of the study are as follows:

*H1:* Desire for being liked is negatively associated with psychological resilience.

*H2:* Desire for being liked is positively associated with smartphone addiction.

*H3:* Smartphone addiction is negatively associated with psychological resilience.

*H4:* Smartphone addiction statistically mediates the association between desire for being liked and psychological resilience.

## 2 Method

### 2.1 Research model

The study employed a quantitative research design based on the correlational survey model. The correlational survey model is one of the research designs used to determine the direction and strength of relationships between two or more variables, allowing the examination of patterns of covariation among variables (Christensen et al., 2015; Karasar, 2023). Because the constructs investigated in the social sciences are generally multidimensional and dynamic, evaluating relationships solely through bivariate associations may provide a limited understanding of the interrelationships among variables. In this context, mediation analysis allows researchers to examine whether the association between a predictor and an outcome variable is statistically transmitted through a third variable, thereby providing a more comprehensive assessment of the pattern of associations within a theoretically informed framework. (Figure 1). Accordingly, examining mediating variables can contribute to the empirical evaluation of theoretically proposed relationships among variables, while the resulting mediation effects should be interpreted as statistical associations rather than causal mechanisms in cross-sectional research designs (Gürbüz, 2021).

**Figure 1:**
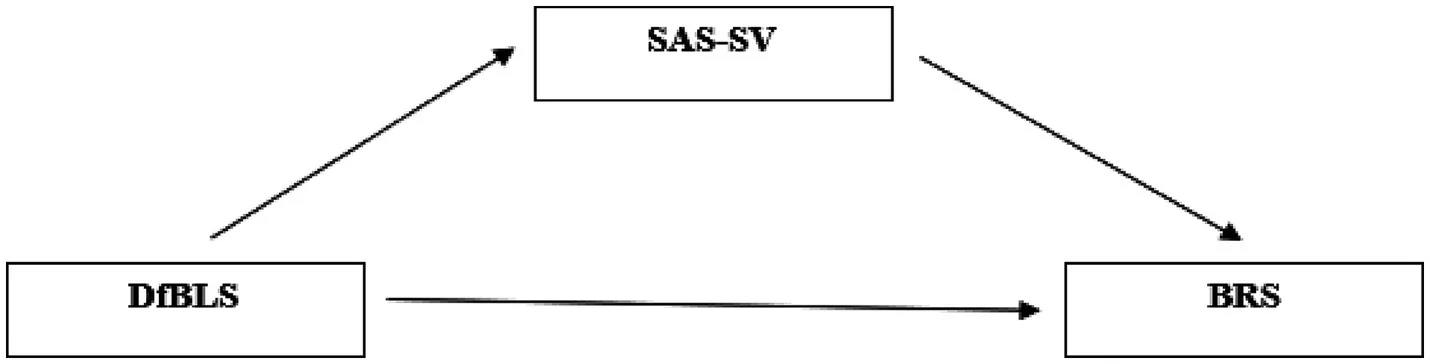
Hypothesized cross-sectional mediation model. DfBLS, desire for being liked scale; SAS-SV, smartphone addiction scale–short version; BRS, brief resilience scale; Arrows indicate hypothesized associations.

### 2.2 Participants

A cross-sectional study was conducted among undergraduate students enrolled in faculties of sport sciences at public universities in Türkiye. Participants were recruited using a convenience sampling strategy based on voluntary participation (Cohen et al., 2011). Eligibility was limited to undergraduate students who were currently enrolled in a faculty of sport sciences, were at least 18 years of age, and voluntarily agreed to participate. Individuals who did not meet these eligibility criteria or who chose not to participate were excluded from the study. Before data collection, *a priori* power analysis was conducted using G*Power 3.1 (Faul et al., 2009). The analysis was performed using the *F*-test family and the Linear multiple regression: Fixed model, R^2^ deviation from zero procedure. Assuming a medium effect size (Cohen’s f^2^ = 0.15), a significance level of *α* = 0.05, a statistical power (1 − β) of 0.95, and two predictors, the minimum required sample size was estimated to be 107 participants. The final sample consisted of 237 participants, which exceeded the required sample size and therefore provided adequate statistical power for the planned analyses. Ethical approval for the study was obtained on 4 June 2026, and data collection was conducted only after ethical approval had been granted, between 5 June and 15 June 2026. Data were collected online using Google Forms. The survey link was distributed to 500 undergraduate students enrolled in faculties of sport sciences at public universities in Türkiye. Each participant was permitted to complete the questionnaire only once. A total of 237 students voluntarily completed the survey, whereas 263 invited students did not respond, yielding a response rate of 47.4%. Because all questionnaire items were set as mandatory, no questionnaires were excluded due to missing or incomplete responses. The final sample consisted of 237 undergraduate students (159 males and 78 females) with a mean age of 21.80 years (SD = 2.13; range = 18–37 years). Male participants had a mean age of 22.03 years (SD = 2.26; range = 18–37), whereas female participants had a mean age of 21.32 years (SD = 1.76; range = 18–26). Overall, the sample represented a young adult university population.

### 2.3 Data collection instruments

#### 2.3.1 Desire for being liked scale

The Desire for Being Liked Scale, developed by Kaşıkara and Doğan (2017), is a unidimensional instrument consisting of nine items rated on a four-point Likert scale. The scale contains no reverse-coded items, and higher scores indicate a stronger desire for being liked. In the original scale development study, exploratory factor analysis showed that the single-factor structure explained 42% of the total variance, and Confirmatory Factor Analysis (CFA) supported this factorial structure. Cronbach’s alpha coefficients were reported as 0.82 for the exploratory factor analysis sample and 0.81 for the CFA sample.

Because the data demonstrated no substantial deviations from normality, CFA was conducted using the Maximum Likelihood estimation method. The initial CFA indicated that the measurement model did not demonstrate an acceptable fit to the data (CMIN/df = 4.826, GFI = 0.890, CFI = 0.899, TLI = 0.866, RMSEA = 0.127, SRMR = 0.055). Therefore, modification indices (MIs) were examined, and only theoretically justifiable correlated residuals between items within the same latent construct were specified. First, a covariance was specified between the error terms of Items 1 and 2 (e1 ↔ e2; MI = 24.049), as both items share highly similar content referring to negative emotional reactions following the absence of social approval, particularly feelings of worthlessness and disappointment when approval is not received. Second, a covariance was added between the error terms of Items 2 and 8 (e2 ↔ e8; MI = 14.584), because both items address closely related emotional consequences of not being liked, including disappointment and concerns about social exclusion. Finally, a covariance was specified between the error terms of Items 1 and 5 (e1 ↔ e5; MI = 14.554), as both items reflect emotional vulnerability associated with anticipated rejection and emphasize feelings of worthlessness and anxiety about not being liked. No correlated residuals were specified between items belonging to different latent constructs, and no additional modifications were introduced beyond those supported by both the modification indices and substantive theoretical considerations. Following these modifications, the final measurement model demonstrated an acceptable fit to the data (CMIN = 71.542, df = 24, CMIN/df = 2.981, TLI = 0.931, CFI = 0.954, RMSEA = 0.080, SRMR = 0.0415) (Baumgartner and Homburg, 1996; Hair et al., 2013; Kline and Little, 2023). Furthermore, convergent validity was evaluated through the Average Variance Extracted (AVE) and Composite Reliability (CR) values. Although the AVE value (AVE = 0.493) was slightly below the conventional threshold of 0.50, the high composite reliability value (CR = 0.897) and satisfactory standardized factor loadings supported the adequacy of the measurement model (Fornell and Larcker, 1981; Hair et al., 2021). In the present study, the internal consistency of the scale was supported by both Cronbach’s alpha (*α* = 0.89) and McDonald’s omega (*ω* = 0.896), indicating good reliability (Table 1).

**Table 1:** Goodness of fit indices and threshold values used in CFA, Reliability, and normality analyses.

Index	Good fit	Acceptable	DfBLS	SAS-SV	BRS
X^2^/df	<3	<3(X^2^/df) < 5	2.981	2.548	1.051
TLI	>0.95	>0.90	0.931	0.936	0.998
CFI	>0.95	>0.90	0.954	0.954	0.999
RMSEA	<0.05	<0.08	0.080	0.077	0.015
SRMR	<0.05	<0.08	0.041	0.050	0.020
Cronbach alpha	0.90	0.60–0.80	0.89	0.88	0.70
McDonald’s omega	0.80–0.89	0.70–0.79	0.896	0.894	0.692
Skewness	-	−1.5/+1.5	0.232	−0.090	−0.110
Kurtosis	-	−1.5/+1.5	−0.335	−0.729	1.328
AVE	>0.50	>0.40	0.493	0.459	0.422
CR	>0.70	>0.70	0.897	0.893	0.806

DfBLS, desire for being liked scale; SAS-SV, smartphone addiction scale-short version; BRS, the brief resilience scale; *χ*^2^/df, chi-square/degrees of freedom; TLI, Tucker–Lewis index; CFI, comparative fit index; RMSEA, root mean square error of approximation; SRMR, standardized root mean square residual; *α*, Cronbach’s alpha; *ω*, McDonald’s omega; AVE, average variance extracted; CR, composite reliability.

#### 2.3.2 Smartphone addiction scale–short version

Problematic smartphone use was assessed using the Smartphone Addiction Scale–Short Version (SAS-SV) developed by Kwon et al. (2013). The scale is a unidimensional instrument consisting of 10 items rated on a six-point Likert scale. Higher scores indicate greater levels of problematic smartphone use (i.e., more severe smartphone addiction symptoms). The SAS-SV is intended to assess the severity of problematic smartphone use symptoms rather than to provide a clinical diagnosis of smartphone addiction. In the original validation study, the scale demonstrated a Cronbach’s alpha coefficient of 0.91, whereas the Turkish adaptation by Noyan et al. (2015) reported a coefficient of 0.867.

In the present study, CFA was conducted using the Maximum Likelihood estimation method. The initial CFA indicated that the measurement model did not demonstrate an acceptable fit to the data (CMIN/df = 4.509, GFI = 0.867, CFI = 0.886, TLI = 0.854, RMSEA = 0.122, SRMR = 0.0645). Therefore, modification indices (MIs) were examined, and only theoretically justifiable correlated residuals between items within the same latent construct were specified. First, a covariance was specified between the error terms of Items 1 and 2 (e1 ↔ e2; MI = 43.844), as both items assess functional impairment resulting from excessive smartphone use, particularly difficulties in fulfilling planned tasks and academic or work-related responsibilities. Second, a covariance was added between the error terms of Items 4 and 10 (e4 ↔ e10; MI = 15.277), because both items reflect excessive smartphone involvement that is evident to both the individual and others, representing behavioral manifestations of problematic smartphone use. Finally, a covariance was specified between the error terms of Items 5 and 9 (e5 ↔ e9; MI = 8.897), as both items capture diminished self-regulation associated with smartphone use, including emotional discomfort when separated from the smartphone and difficulties controlling the duration of smartphone use. No correlated residuals were specified between items belonging to different latent constructs, and no additional modifications were introduced beyond those supported by both the modification indices and substantive theoretical considerations. Following these modifications, the final measurement model demonstrated an acceptable fit to the data (CMIN = 81.526, df = 32, CMIN/df = 2.548, TLI = 0.936, CFI = 0.954, RMSEA = 0.077, SRMR = 0.0508) (Baumgartner and Homburg, 1996; Hair et al., 2013; Kline, 2023). The convergent validity assessment indicated an AVE value of 0.459 and a CR value of 0.893. Although the AVE value was below the recommended threshold of 0.50, the high composite reliability coefficient and standardized factor loadings provided evidence supporting the adequacy of the measurement model (Fornell and Larcker, 1981; Hair Jr et al., 2021). In the present study, the internal consistency of the scale was supported by Cronbach’s alpha (*α* = 0.88) and McDonald’s omega (*ω* = 0.894), indicating good reliability (Table 1).

#### 2.3.3 Brief resilience scale

Psychological resilience was operationalized as individuals’ perceived ability to recover or “bounce back” from stress using the Brief Resilience Scale (BRS) developed by Smith et al. (2008) and adapted into Turkish by Doğan (2015). The scale consists of six items with a single-factor structure rated on a five-point Likert scale. Items 2, 4, and 6 are reverse scored before calculating the total score. Higher scores indicate a greater perceived ability to recover or bounce back from stressful experiences. In the Turkish adaptation study, Doğan (2015) reported an internal consistency coefficient of 0.83 and a Cronbach’s alpha coefficient of 0.81, supporting the reliability and validity of the scale for use in the Turkish population. In the present study, CFA was conducted using the Maximum Likelihood estimation method. The initial CFA indicated that the measurement model did not demonstrate an acceptable fit to the data (CMIN/df = 12.531, GFI = 0.852, CFI = 0.695, TLI = 0.492, RMSEA = 0.221, SRMR = 0.1412). Therefore, modification indices (MIs) were examined, and only theoretically justifiable correlated residuals between items within the same latent construct were specified. First, a covariance was specified between the error terms of Items 1 and 5 (e1 ↔ e5; MI = 44.000), as both items assess the perceived ability to recover quickly and effectively from adverse experiences. Second, a covariance was added between the error terms of Items 1 and 3 (e1 ↔ e3; MI = 20.284), because both items reflect rapid psychological recovery following stressful situations. Finally, a covariance was specified between the error terms of Items 3 and 5 (e3 ↔ e5; MI = 23.215), as both items assess resilience in terms of recovering from stress with limited difficulty and within a relatively short period. No correlated residuals were specified between items belonging to different latent constructs, and no additional modifications were introduced beyond those supported by both the modification indices and substantive theoretical considerations. Following these modifications, the final measurement model demonstrated an excellent fit to the data (CMIN = 6.304, df = 6, CMIN/df = 1.051, TLI = 0.998, CFI = 0.999, RMSEA = 0.015, SRMR = 0.0201) (Baumgartner and Homburg, 1996; Hair et al., 2013; Kline, 2023). The convergent validity assessment yielded an AVE value of 0.422 and a CR value of 0.806. Although the AVE value did not reach the conventional threshold of 0.50, the composite reliability coefficient exceeded the recommended level and the standardized factor loadings provided additional evidence regarding the adequacy of the measurement model (Fornell and Larcker, 1981; Hair Jr et al., 2021). In the present study, Cronbach’s alpha (*α* = 0.70) and McDonald’s omega (*ω* = 0.692) indicated acceptable internal consistency for research purposes; however, these coefficients were interpreted with appropriate caution because they were close to the lower threshold of acceptable reliability (Table 1).

### 2.4 Ethical approval

This study was reviewed and approved by the Human Research, Health and Sports Sciences Ethics Committee of Erzincan Binali Yıldırım University (Decision No. 05/11), issued during its fifth session held on June 4, 2026. The research was conducted in accordance with the ethical principles outlined in the Declaration of Helsinki for research involving human participants.

### 2.5 Statistical analysis

Before the statistical analysis, the data were screened using IBM SPSS Statistics 25 to examine missing data, outliers, and the assumption of normality. As no outliers were identified, all analyses were conducted using the data obtained from 237 students enrolled in the faculties of sports sciences. The assumption of normality was evaluated based on skewness and kurtosis coefficients, with *Z*-scores and Mahalanobis distances also taken into consideration. The skewness and kurtosis values of all variables fell within the ±1.5 range, indicating that the assumption of normality was satisfied (Tabachnick and Fidell, 2013). Furthermore, Maximum Likelihood (ML) estimation was employed for the confirmatory factor analyses because the item distributions showed no substantial deviations from normality. In addition, previous methodological studies have demonstrated that ML estimation provides robust parameter estimates when Likert-type items include four or more response categories and the sample size is adequate (Flora and Curran, 2004; Rhemtulla et al., 2012). Pearson correlation analysis was performed to examine the relationships among the predictor and outcome variables. To evaluate the construct validity of the measurement instruments, CFAs were conducted using IBM AMOS 24. After the measurement models had been validated, the research hypotheses were tested using regression analyses based on the bootstrap procedure (Hayes, 2022). The mediating role of smartphone addiction was examined using PROCESS Macro Model 4. Because of the unequal distribution of male and female participants in the sample, participant gender was included as a covariate in the mediation analyses to account statistically for potential differences associated with gender. The inclusion of gender did not materially alter the direction or statistical significance of the direct and indirect effects. To obtain more robust parameter estimates, a bootstrap procedure with 5,000 resamples was employed (Hayes et al., 2017). The significance of the indirect effect was determined based on whether the 95% confidence interval excluded zero (Preacher and Hayes, 2008; Hayes, 2022). In addition, Cronbach’s alpha coefficients of 0.70 or higher were considered indicative of acceptable internal consistency (Nunnally and Bernstein, 1994). Consistent with this interpretation, Kalaycı (2018) suggested that Cronbach’s alpha values between 0.60 and 0.80 indicate a reasonably reliable scale, whereas values above 0.80 indicate high reliability. McDonald’s omega coefficients were also calculated to provide a complementary estimate of internal consistency, with values of 0.70 or higher considered acceptable and values of 0.80 or higher indicating good reliability (Hayes and Coutts, 2020).

### 2.6 Common method Bias assessment

To minimize the risk of common method bias (CMB), several procedural remedies recommended by Podsakoff et al. (2003) were implemented. Participation was voluntary and anonymous; respondents were informed that there were no right or wrong answers, and all responses were treated confidentially in order to reduce evaluation apprehension and social desirability bias. In addition, validated measurement instruments with established psychometric properties were employed. As a statistical assessment of CMB, Harman’s single-factor test was conducted following the recommendations of Podsakoff et al. (2003). Principal Axis Factoring (PAF) extracted five factors with eigenvalues greater than 1, and the first unrotated factor accounted for 27.40% of the total variance, which is well below the commonly used threshold of 50%. Accordingly, no single factor accounted for the majority of the covariance among the measured variables, suggesting that common method bias is unlikely to represent a serious threat to the validity of the findings.

### 2.7 Discriminant validity

To examine whether desire for being liked, problematic smartphone use, and psychological resilience represented empirically distinct constructs, a three-factor CFA was conducted. The measurement model included all study variables simultaneously, with each item loading onto its respective latent construct. The theoretically justified residual covariances identified during the individual CFA analyses were retained in the combined measurement model because they represented shared variance among conceptually related items within the same constructs. The three-factor measurement model demonstrated an acceptable fit to the data (χ^2^ = 454.003, df = 263, χ^2^/df = 1.726, CFI = 0.927, TLI = 0.917, RMSEA = 0.055, 90% CI [0.047, 0.064], SRMR = 0.063), providing evidence that the proposed three-factor structure adequately represented the observed data.

Discriminant validity was further evaluated using the Fornell–Larcker criterion (Fornell and Larcker, 1981). Specifically, the square root of the Average Variance Extracted (AVE) for each latent construct was compared with its correlations with the remaining constructs. The square roots of the AVE values were 0.702 for Desire for Being Liked, 0.677 for Problematic Smartphone Use, and 0.650 for Psychological Resilience. These values exceeded the corresponding latent inter-construct correlations (|r| = 0.394–0.507), indicating adequate discriminant validity. The latent correlations were 0.394 between desire for being liked and problematic smartphone use, −0.507 between desire for being liked and psychological resilience, and −0.344 between problematic smartphone use and psychological resilience. Collectively, these findings indicate that the three constructs are empirically distinguishable despite being theoretically related (see Table 2).

**Table 2:** Discriminant validity based on the Fornell–Larcker criterion.

Construct	1	2	3
1. Desire for being liked	**0.702**		
2. Smartphone addiction	0.394	**0.677**	
3. Brief resilience	−0.507	−0.344	**0.650**

Diagonal values (bold) represent the square roots of the Average Variance Extracted (AVE). Off-diagonal values represent latent correlations among the constructs.

## 3 Results

The correlation coefficients and mean scores for the study variables, namely desire for being liked, smartphone addiction, and psychological resilience, among students enrolled in the faculties of sports sciences, are presented in Table 3.

**Table 3:** Correlations between variables and descriptive analyses.

Variables	DfBLS	SAS-SV	BRS	Sd	X̄
DfBLS	1	**0.369** ^**^	**−0.264** ^**^	5.82	18.41
SAS-SV		1	**−0.256** ^**^	11.26	32.21
BRS			1	4.06	19.54

^**^p < 0.01 *N* = 237; DfBLS, desire for being liked scale; SAS-SV, smartphone addiction scale-short version; BRS, the brief resilience scale; Sd, standard deviations; CI, confidence interval.

Table 3 presents the descriptive statistics (*M* = mean, SD = standard deviation) and Pearson correlation coefficients among the study variables, namely desire for being liked (DfBLS), Smartphone Addiction (SAS-SV), and Brief Resilience (BRS). The results revealed a moderate positive correlation between desire for being liked and Smartphone Addiction (*r* = 0.369, 95% CI [0.254, 0.474], *p* < 0.001). In addition, desire for being liked was negatively associated with Brief Resilience (*r* = −0.264, 95% CI [−0.379, −0.141], *p* < 0.001). Similarly, Smartphone Addiction showed a negative association with Brief Resilience (*r* = −0.256, 95% CI [−0.371, −0.133], *p* < 0.001). According to conventional guidelines for interpreting correlation coefficients, these findings indicate small-to-moderate to moderate associations among the study variables rather than strong relationships (Cohen, 1988).

According to the results presented in Table 4 and Figure 2, the total association between desire for being liked and brief resilience was negative and statistically significant (*B* = −0.184, 95% CI [−0.271, −0.098], *p* < 0.001, *R*^2^ = 0.070). The effect of desire for being liked on smartphone addiction was positive and statistically significant (*B* = 0.713, *p* < 0.001, *R*^2^ = 0.136). In addition, smartphone addiction was negatively and significantly associated withbrief resilience (*B* = −0.066, *p* = 0.006, *R*^2^ = 0.099). The mediation analysis further indicated a statistically significant indirect association between desire for being liked and brief resilience through smartphone addiction (*B* = −0.047, 95% CI [−0.094, −0.011]). Because the 95% confidence interval did not include zero, the results were consistent with a statistically significant indirect association between desire for being liked and brief resilience through smartphone addiction.

**Table 4:** Analysis results regarding the mediation test using Model 4.

Stage	PV	OV	*B*	SE	*t*	*F*	df	Adj. *R*^2^	*p*	%95 CI [LL, UL]	*β*	*R* ^2^
a path	DfBL	SAS-SV	0.713	0.117	6.09	37.11	1,235	0.133	<0.001	0.483, 0.944	0.369	0.136
b path	SAS-SV	BRS	−0.066	0.024	−2.75	12.83	2,234	0.091	0.006	−0.114, −0.019	−0.184	0.099
Direct Effect (c’ path)	DfBL	BRS	−0.137	0.047	−2.94	12.83	2,234	0.091	0.004	−0.229, −0.045	−0.196	0.099
Total Effect (c path)	DfBL	BRS	−0.184	0.044	−4.20	17.60	1,235	0.066	<0.001	−0.271, −0.098	−0.264	0.070
İndirect Effect (a × b)	DfBL → SAS-SV → BRS	−0.047	0.021						−0.094, −0.011			

*N* = 237; DfBL, desire for being liked; SAS-SV, smartphone addiction scale–short version; BRS, brief resilience scale; PV, prediction variable; OV, outcome variable; B, unstandardized regression coefficient; β, standardized regression coefficient; SE, standard error; *F*, *F* statistic; df, degrees of freedom; *R*^2^, coefficient of determination; Adj. *R*^2^, adjusted coefficient of determination; LLCI, lower limit of the 95% confidence interval; ULCI, upper limit of the 95% confidence interval. The indirect effect was evaluated using a bias-corrected bootstrap procedure (5,000 resamples); therefore, no *t*-value or *p*-value is reported for the indirect effect.

**Figure 2:**
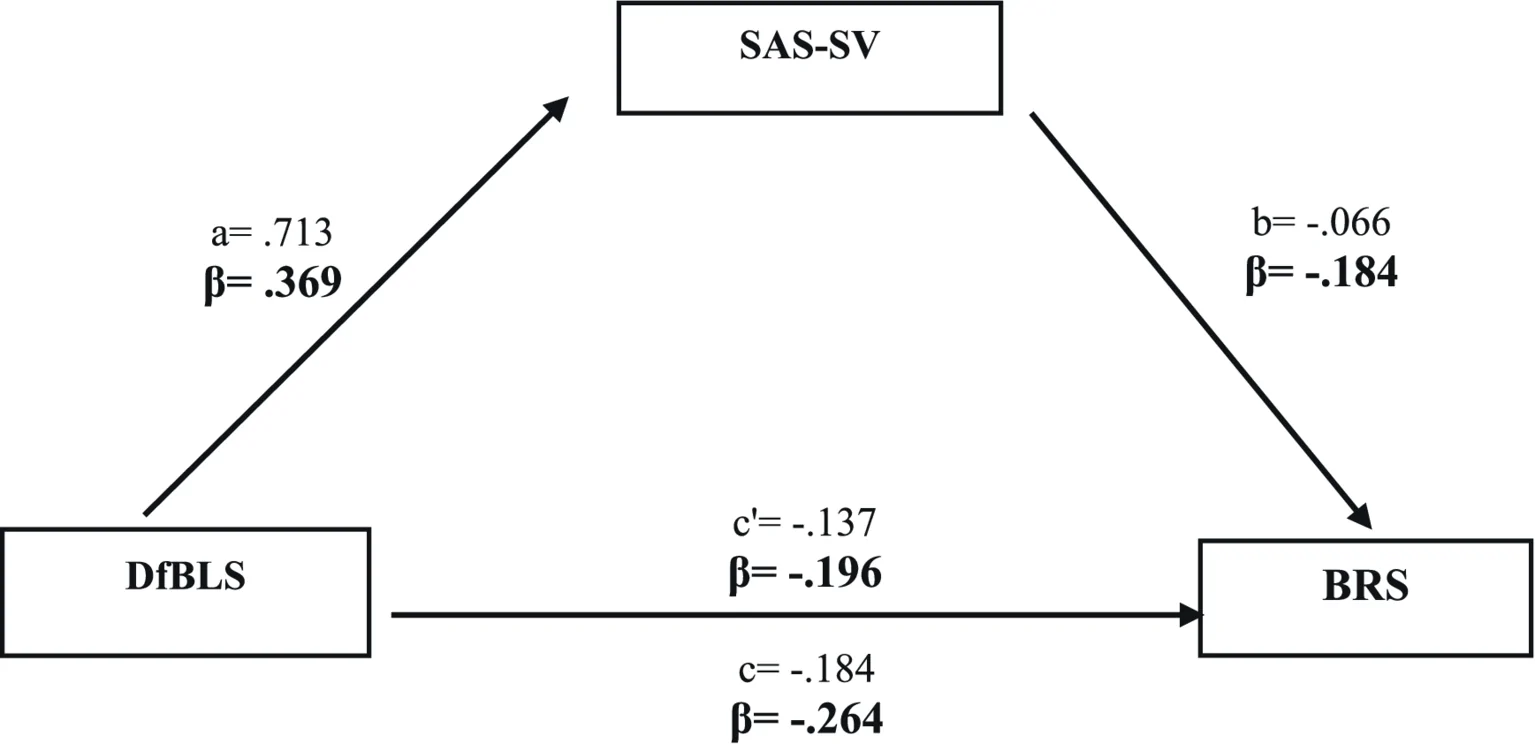
Results of mediation analysis.

## 4 Discussion

The present study examined the mediating role of smartphone addiction in the relationship between the desire to be liked and psychological resilience among students enrolled in sports science faculties. The findings demonstrated that the proposed mediation model was statistically significant and provided a theoretically coherent framework for explaining the relationships among the study variables. The results further suggest that a greater need for social approval and acceptance was associated with higher levels of problematic smartphone use, which was in turn associated with lower psychological resilience. Within the theoretical framework adopted in the present study, smartphone addiction may provide one theoretically plausible interpretation of the observed association between the desire for being liked and psychological resilience. However, because of the cross-sectional design, this interpretation should not be regarded as evidence of temporal or causal relationships.

The first major finding of the study indicated that the desire for being liked was significantly and negatively associated with psychological resilience (H_1_). This finding indicates that greater levels of the desire for being liked were associated with lower levels of psychological resilience in the present sample. It should also be noted that the Desire for Being Liked Scale measures a general tendency to seek social approval rather than approval specifically within digital environments. Therefore, the interpretation of the present findings in relation to digital contexts should be considered theoretical and grounded in the proposed conceptual framework rather than in the measurement instrument itself. From the perspective of SDT (Deci and Ryan, 2000), reliance on external feedback and social approval has been theoretically linked to greater sensitivity to social evaluation (Brunet and Sabiston, 2009; Neighbors and Knee, 2003). From this perspective, dependence on external validation may be relevant to individual differences in intrinsic motivational resources and psychological adjustment. Consistent with the present findings, Civitci and Civitci (2015) reported that university students with higher levels of social approval needs exhibited lower levels of psychological resilience. In the context of sports sciences students, where sport performance, physical appearance, and social evaluation are particularly salient within both academic and social environments, a stronger orientation toward external approval rather than intrinsic development may be associated with lower psychological resilience (Ntoumanis and Biddle, 1998). In addition, previous literature has linked perceived pressure associated with evaluation and approval to performance anxiety and psychological distress, which may be relevant to psychological resilience (Stoeber and Hotham, 2013). However, performance anxiety was not directly assessed in the present study.

Another important finding revealed that the desire to be liked was positively associated with smartphone addiction (H_2_). This finding can be interpreted within the frameworks of SDT (Deci and Ryan, 2000) and the Uses and Gratifications Theory (Katz et al., 1973). According to SDT, relatedness, one of the three basic psychological needs, reflects individuals’ need to be accepted and approved by their social environment (Deci and Ryan, 2008). Digital media environments facilitate the satisfaction of this need by providing immediate feedback, visibility, and opportunities for social interaction (Zhu and Chen, 2015). Consequently, individuals with stronger needs for social acceptance and approval may be more inclined toward intensive smartphone use (Panek et al., 2018). From the perspective of the Uses and Gratifications Theory, individuals actively use digital media to satisfy psychological needs such as social interaction, a sense of belonging, and social approval (Aksoy and Allahverdi, 2025). However, persistent engagement with these platforms has been associated with loss of control and behavioral addiction patterns in previous research. Previous studies involving university students have similarly shown that the risk of smartphone addiction increases as the need for social approval becomes stronger (Long et al., 2016). Likewise, Aladwani and Almarzouq (2016) argued that social media interactions function as a form of digital reward that reinforces addictive tendencies. Among sports sciences students, the salience of athletic identity and physical appearance in these contexts may provide a relevant contextual perspective for understanding the pursuit of social approval in digital environments.

The finding that smartphone addiction was negatively associated with psychological resilience (H_3_) may be interpreted within the framework of Social Comparison Theory. According to this theory, individuals tend to compare themselves with others to evaluate their own abilities and life circumstances (Festinger, 1954). From the perspective of Social Comparison Theory, digital media environments may provide opportunities for individuals to encounter idealized representations of others’ lifestyles, achievements, and physical appearances (Vogel et al., 2014). In sports sciences contexts, such content may include athletic physiques, training routines, and sport performance. However, social comparison processes and exposure to these specific forms of content were not directly assessed in the present study. Arroyo and Brunner (2016) reported that exposure to fitness-related content on digital platforms may be associated with social comparison processes and negative body-related evaluations. These findings provide a possible contextual interpretation; however, neither social comparison nor body-related evaluations were directly measured in the present study. Such experiences may be associated with greaterfeelings of inadequacy, stress, and psychological distress, particularly among individuals with intensive smartphone use (Augner and Hacker, 2012). Similarly, Hou et al. (2017) and Raj et al. (2023) reported that problematic technology use has been associated with lower cognitive flexibility and poorercapacity to recover from stressful life events. These findings may provide a possible theoretical context for understanding the observed association between smartphone addiction and psychological resilience, although cognitive flexibility was not directly assessed in the present study. Accordingly, digital addiction processes be associated with differencest the cognitive and emotional resources that are essential for maintaining psychological resilience. Within the context of sports sciences students, digital content related to physical performance and athletic achievement may constitute a relevant contextual factor when considering the broader literature on social comparison. However, body image, athletic achievement, and social comparison were not directly assessed in the present study. Accordingly, continuous online engagement and digital interaction pressures may be associated with lower psychological resilience. As previously suggested, psychological resilience represents an important psychosocial determinant of life satisfaction among sports sciences students (Secer et al., 2025).

The mediation analysis indicated a statistically significant indirect association between the desire for being liked and psychological resilience through smartphone addiction (H_4_). The observed pattern is consistent with the possibility that stronger needs for social approval may be associated with greater engagement in digital interactions, while intensive and uncontrolled smartphone use may also be associated with lower psychological resilience. When interpreted collectively within the frameworks of SDT, the Uses and Gratifications Theory, and Social Comparison Theory, the findings suggest that the pursuit of social approval may be relevant tobehavioral addiction patterns, while problematic smartphone use may be associated with differences in psychological adjustment capacity (Leary and Gabriel, 2022). Consistent with this interpretation, Dhir et al. (2018) reported that problematic technology use may negatively affect individuals’ psychological wellbeing.

The mediation findings further indicate a significant indirect association between the desire for being liked and psychological resilience through smartphone addiction, although this indirect association did not fully account for the overall association. This finding suggests that the direct statistical association represented by the direct effect remained significant, indicating that the desire for social approval may independently influence individuals’ psychological resilience beyond digital addiction processes. Among students in sports sciences, the central role of physical performance and social visibility within academic and social contexts may provide a relevant contextual perspective for understanding the association between approval-seeking and psychological resilience.

Although the proposed mediation model was statistically significant, the observed correlations and explained variance were modest. Specifically, the model explained 13.6% of the variance in problematic smartphone use and 9.9% of the variance in psychological resilience. These findings suggest that, although the desire for being liked and problematic smartphone use are meaningful correlates of psychological resilience, a substantial proportion of the variance remains unexplained. Psychological resilience is a multidimensional construct associated with a wide range of individual, interpersonal, and contextual factors, including personality characteristics, coping strategies, perceived social support, and life experiences (Smith et al., 2008). Therefore, future studies should incorporate additional psychological and environmental variables to develop more comprehensive explanatory models and improve the explanatory power of the proposed mediation framework. Moreover, alternative temporal explanations cannot be ruled out. For example, lower psychological resilience may be associated with greater vulnerability to problematic smartphone use, which may also be associated with stronger approval-seeking tendencies. Longitudinal and experimental studies are therefore needed to clarify the temporal ordering of these relationships. Furthermore, the AVE values obtained for the measurement instruments were below the conventional 0.50 criterion. Although composite reliability values were satisfactory and the factor structures were supported by confirmatory factor analyses, the convergent validity findings should be interpreted with appropriate caution. Similarly, the internal consistency of the Brief Resilience Scale was at the lower acceptable boundary, suggesting that findings involving psychological resilience should be interpreted cautiously and replicated using alternative resilience measures. Although Harman’s single-factor test did not indicate substantial common method bias, the exclusive reliance on self-report measures collected at a single time point means that common method variance cannot be entirely ruled out.

### 4.1 Limitations

Several limitations should be considered when interpreting the findings of this study. First, the cross-sectional design limits causal inferences regarding the relationships among the study variables. Although the mediation model was statistically significant, the temporal ordering implied by the proposed mediation model could not be established. Therefore, the findings should be interpreted as evidence of statistical mediation within a correlational framework rather than as confirmation of a temporal or causal mediation process. Moreover, alternative directional models cannot be ruled out, and future longitudinal and experimental studies are needed to clarify the temporal sequence and causal direction of the observed associations. Second, convenience sampling was employed, which limits the generalizability of the findings and may have introduced selection bias. In addition, although the response rate has been reported in the Methods section, participants were recruited exclusively from faculties of sports sciences in Türkiye, limiting the representativeness of the sample. Another limitation is that the study was conducted exclusively with students enrolled in faculties of sports sciences, and the gender distribution of the sample was not balanced. This imbalance largely reflects the differing admission quotas for male and female students across sports sciences programs. Accordingly, future studies are encouraged to replicate these findings using more diverse samples. Furthermore, although participant gender was statistically controlled in the mediation analyses, several potentially important confounding variables—including socioeconomic status, actual smartphone use duration, social media use, mental health symptoms, physical activity level, and year of study—were not measured and therefore could not be included in the analyses. Consequently, residual confounding cannot be completely ruled out, and future studies should incorporate these variables to provide more precise estimates of the observed relationships. Another limitation concerns the measurement model. Although the confirmatory factor analyses demonstrated acceptable overall model fit after theoretically justified residual covariances were specified, these post-hoc modifications were identified and evaluated within the same sample and therefore should be interpreted cautiously until replicated in independent samples. In addition, although convergent and discriminant validity were considered acceptable overall, the AVE values were below the conventional 0.50 criterion, and the Brief Resilience Scale demonstrated reliability coefficients close to the conventional lower threshold for acceptable internal consistency. These findings suggest that the psychometric properties of the measures should be interpreted with appropriate caution.

The proposed mediation model also demonstrated modest explanatory power, indicating that substantial variance in problematic smartphone use and psychological resilience remains unexplained. Future studies should incorporate additional psychological, behavioral, and contextual variables to develop more comprehensive explanatory models. Moreover, because the present study assessed a general desire for being liked rather than digital-specific approval-seeking and did not directly measure social media use behaviors or online approval-seeking, future research should include these constructs to better understand the mechanisms underlying digital technology use. Finally, because the data were collected through self-report measures at a single time point, the findings may have been influenced by social desirability bias, subjective reporting, and common method variance. Although Harman’s single-factor test suggested that common method bias was unlikely to represent a serious threat, more rigorous approaches (e.g., marker-variable or latent method factor techniques) and multi-method data collection strategies are recommended for future research.

### 4.2 Practical implications and conclusion

The findings highlight the need to promote healthy digital media practices within educational settings and to increase awareness of the potential adverse effects of digital addiction on mental health (Kaur and Mehndroo, 2024). Although the present cross-sectional study did not evaluate the effectiveness of specific interventions, the observed associations may provide a useful basis for designing and evaluating future digital wellbeing initiatives among students in faculties of sports sciences. In particular, awareness programs addressing smartphone use, psychoeducational interventions designed to enhance self-regulation skills, and initiatives promoting healthy digital use habits may represent promising directions for future intervention research.

From a theoretical perspective, the findings contribute to the growing literature on digital wellbeing by integrating SDT, the Uses and Gratifications Theory, and Social Comparison Theory into a single explanatory framework. This integrated perspective highlights how individual motivational characteristics and digital behaviors may be associated with psychological resilience in young adults.

From an applied perspective, the findings suggest that promoting healthy technology use could be considered as one component of student wellbeing initiatives in higher education, particularly within faculties of sports sciences. However, longitudinal and experimental studies are needed to determine whether interventions targeting social approval-seeking, self-regulation, or smartphone use produce measurable improvements in psychological resilience.

In addition, sport psychology professionals and academic advisors may wish to be aware of students’ technology use motivations, social approval-seeking tendencies, and patterns of digital interaction as potential correlates of psychological wellbeing. Future research should determine whether these factors represent appropriate targets for preventive or educational interventions.

In conclusion, the findings suggest that the desire for being liked may be positively associated with smartphone addiction among students in faculties of sports sciences and that higher levels of smartphone addiction may be associated with lower psychological resilience. Furthermore, the results provide evidence that smartphone addiction may function as a statistical mediator in the association between the desire for being liked and psychological resilience. Accordingly, increased social approval-seeking in digital environments may indirectly weaken individuals’ psychological adjustment processes. Because the present study employed a cross-sectional design, these findings should be interpreted as evidence of statistical associations rather than causal effects. Future longitudinal and intervention studies are required to determine whether modifying smartphone use or approval-seeking leads to improvements in psychological resilience.

## Data Availability

The raw data supporting the conclusions of this article will be made available by the authors, without undue reservation.
